# An economic evaluation of pharmacopuncture versus usual care for chronic neck pain: a pragmatic randomized controlled trial

**DOI:** 10.1186/s12913-023-10325-w

**Published:** 2023-11-23

**Authors:** Doori Kim, Eun-San Kim, Hyun Jin Song, Sun-Young Park, Kyoung Sun Park, Yoon Jae Lee, In-Hyuk Ha

**Affiliations:** 1 Jaseng Spine and Joint Research Institute, Jaseng Medical Foundation, 540, Gangnamdae-ro, Gangnam-gu, Seoul, 06110 Republic of Korea; 2 College of Pharmacy, University of Florida, 1225 Center Drive, Gainesville, Florida, FL 32610 USA; 3 VIAplus, 6th floor, 197 Kwongwang-ro, Paldal-gu, Suwon, Gyeonggi-do Republic of Korea; 4 Jaseng Hospital of Korean Medicine, 536, Gangnam-daero, Gangnam-gu, Seoul, 06110 Republic of Korea

**Keywords:** Pharmacopuncture, Neck pain, Musculoskeletal disorder, CAM, Korean medicine, Cost-effectiveness

## Abstract

**Background:**

This study aimed to evaluate the cost utility of pharmacopuncture in comparison with usual care for patients with chronic neck pain.

**Methods:**

A 12-week, multicenter, pragmatic randomized controlled trial was conducted, and 101 patients suffering from chronic neck pain for more than six months were randomly placed into the pharmacopuncture and usual care groups to receive four weeks of treatment and 12 weeks of follow-up observations. The quality-adjusted life year (QALY) was calculated using EQ-5D and SF-6D. Concerning costs in 2019, a primary analysis was performed on societal perspective cost, and an additional analysis was performed on healthcare perspective cost.

**Results:**

Compared to usual care, pharmacopuncture was superior as it showed a slightly higher QALY and a lower incremental cost of $1,157 from a societal perspective. The probability that pharmacopuncture would be more cost-effective at a willingness-to-pay (WTP) of $26,374 was 100%. Pharmacopuncture was also superior from a healthcare perspective, with a lower incremental cost of $26. The probability that pharmacopuncture would be more cost-effective at a WTP of $26,374 was 83.7%.

**Conclusions:**

Overall, pharmacopuncture for chronic neck pain was found to be more cost-effective compared to usual care, implying that clinicians and policy makers should consider new treatment options for neck pain.

**Trial registration:**

Number NCT04035018 (29/07/2019) Clinicaltrials.gov; Number KCT0004243 (26/08/2019) Clinical Research Information Service.

**Supplementary Information:**

The online version contains supplementary material available at 10.1186/s12913-023-10325-w.

## Background

 Neck pain is a common musculoskeletal disorder among the general population, with a one-year prevalence of 15.8% [1], whereas the lifetime prevalence of neck pain among adults is approximately 50% [2]. According to the 2016 Global Burden of Disease [3], neck pain is the fourth main cause of years lived with disability (YLD). It can develop into severe pain and disability in addition to causing a financial burden [4].

Despite various treatment options for neck pain, many patients seek complementary and alternative medicine (CAM) treatment approaches [5], expecting them to be more effective and safer [6]. Pharmacopuncture, which combines traditional acupuncture therapy with herbal medicine, is one of the most popular CAM treatments. It is a method that uses a syringe to inject herbal extracts into acupoints, which maximizes the therapeutic effect of the physical stimulation of acupuncture with the added chemical effect of pharmacopuncture solution [7, 8]. Studies have demonstrated that pharmacopuncture is actively used in Korea to treat musculoskeletal disorders. In one study using a questionnaire survey, it was reported that 118 out of 123 (95.9%) Korean medicine (KM) doctors in spine clinics used pharmacopuncture for spine treatment [9].

However, high-quality evidence of the efficacy of pharmacopuncture is still lacking. According to a systematic review that analyzed the efficacy of pharmacopuncture for cervical spondylosis, pharmacopuncture was found to be more effective in managing pain levels and dysfunction than other controls such as acupuncture and physical therapy (PT); however, the quality of evidence was assessed to be low or very low [10]. A systematic review that analyzed the efficacy of bee venom acupuncture, a type of pharmacopuncture, on musculoskeletal pain reported that it significantly reduced the visual analog scale (VAS) score; however, a firm conclusion could not be drawn due to the small number of RCTs included in the analysis as well as the small sample size [11].

Therefore, a pragmatic RCT was conducted to identify the pharmacopuncture’s efficacy for neck pain [12], and cost-utility analysis from societal perspectives was carried out along with the RCT. The purpose of this study is to provide useful information to clinicians and patients considering treatment options, and policymakers who establish policies on coverage and budget allocation by comparing the cost-effectiveness of pharmacopuncture and usual care based on the real world.

## Methods

### Design

A cost-effectiveness analysis was conducted with a 12-week pragmatic RCT [12]. The results of this clinical trial with the protocol have been published separately [12, 13]. This RCT was briefly conducted alongside a two-armed, parallel, multicenter (four KM Hospitals) RCT that included 101 patients across four institutions in Korea. Enrolled patients were randomly allocated to the pharmacopuncture and PT groups in a 1:1 ratio. The participants received a total of eight treatment sessions for four weeks, followed by eight weeks of follow-up. During this period, pain indicators, quality of life (QOL) indicators, and medical expenditure were investigated.

Schedule of the participants is shown in Supplementary Table A1 (see Additional file 1). The protocol has been registered with Clinicaltrials.gov (Identifier: NCT04035018 (29/07/2019)) and the Clinical Research Information Service (Identifier: KCT0004243 (26/08/2019)). This study followed the Consolidated Standards of Reporting Trials (CONSORT) reporting guidelines.

### Participants

Eligible patients were aged 17–70, had been experiencing neck pain for more than six months, and had a VAS score of more than five for their neck pain. The exclusion criteria for participation were as follows: having cancer metastasis or fracture in the spine; having progressive or severe neurologic deficits or other disorders that may have affected the results; suffering from other types of cancer, rheumatoid arthritis, or stroke; taking medications, such as steroids; being pregnant; having had cervical surgery during the last three months; and facing difficulties participating in the trial as determined by the researcher. The specific inclusion and exclusion criteria can be found in the study conducted by Park et al. [12].

### Intervention

The general rule for pharmacopuncture treatment was undergoing two sessions per week for four weeks; however, one to three sessions per week were permitted depending on the patient’s condition and the physician’s determination. The type of pharmacopuncture solution and treatment points were determined by the physician. All intervention-related matters were recorded in the patient’s electronic medical records (EMR) and case report form (CRF).

The general rules for usual care were similar to those for pharmacopuncture treatment. A Korean Health Insurance Review and Assessment’s (HIRA) analysis claimed that data in a previous study showed that combinations of various types of PT (e.g., superficial heat therapy, deep heat therapy, traction, and electrotherapy) were prescribed [14]. PT was selected based on clinical determination by a physician from those used in previous studies, while the type, duration, and area where all interventions were to be applied were recorded in the EMR and CRF.

### Randomization and blinding

Participants who were determined to be eligible based on the inclusion and exclusion criteria and signed the Informed consent form for this clinical study were assigned to two groups at a ratio of 1:1 using a randomization table. The randomization table was created in advance by a statistician using R studio 1.1.463 (© 2009–2018 RStudio, Inc., Boston, Massachusetts, United States). The random sequence was generated by block randomization, and the size of one block was randomly set between 2, 4 and 6.

The randomization results were sealed in an opaque envelope and delivered to each institution for storage under a double lock. Randomization and assignment of registration numbers for eligible participants was done by opening sealed envelopes. A random number assigned to each participant was recorded on an electronic chart. Due to the nature of our intervention, only evaluators were blinded to group assignments. The evaluator performed outcome evaluation in a separate space before intervention.

### Tools

Both EQ-5D and SF-12 scores were measured at baseline and at intervals of 5, 8, and 12 weeks after randomization. The Korean version of the EQ-5D with verified validity was used, and utility was calculated by applying the tariffs used by Kim et al. [15]. In the trial, a 12-item short-form health survey version 2 (SF-12v2) was used to assess QOL, and the values obtained by SF-12v2 were converted to SF-6D values using the equation described by Brazier et al. [16]. The QALY was calculated using the area under the curve (AUC) approach and trapezoidal rules, using regression analyses adjusted for baseline values [17].

### Unit costs

Supplementary Table A2 (see Additional file 1) shows the sources and unit costs of the interventions applied during the trial. Because the cost of pharmacopuncture intervention may vary across hospitals, as it is a non-reimbursed item, the fees published on various hospitals’ websites were surveyed, and a cost of 20,000 won (around $17) per session was derived. Costs of examination and PT were obtained from the 2019 HIRA Health Insurance Medical Care Benefit Expenses data [18]. The costs were calculated using the type and frequency of treatments prescribed to the participants. Among the types of PT used, the non-reimbursed items’ cost not covered by health insurance was determined by investigating the costs generally prescribed by medical institutions.

### Resource use measurement

In addition to the intervention used in the trial, healthcare services used personally by the patients for neck pain were investigated at baseline and at 5, 8, and 12 weeks using a questionnaire. The questionnaire was used to survey the non-reimbursement and copayments made by the patients as well as the frequency of use. Payer reimbursement was calculated by gender and age by analyzing the records for cervicalgia (Korean Standard Classification of Diseases code: M54.2) in the 2018 HIRA-National Patient Samples (HIRA-NPS) data [19].

The transportation costs were determined through a questionnaire survey. The time spent by patients to receive treatment was surveyed, and the time cost was calculated using the time spent by each individual based on the standard wages for their corresponding gender and age given in the 2019 Survey Report on Labor Conditions by Employment Type [20].

Productivity loss was investigated using the Work Productivity and Activity Impairment – Specific Health Problem (WPAI-SHP) questionnaire [21] at baseline and 2, 3, 4, 5, 8, and 12 weeks after randomization. The WPAI-SHP is used to measure absenteeism (missed work time), presenteeism (impairment while working), overall productivity loss (absenteeism + presenteeism), and activity impairment (impairment in regular activities) in the past week due to a specific health problem (i.e., chronic neck pain) [22, 23]. The present study attempted to account for not only productivity loss among waged workers but also the productivity loss and opportunity cost associated with health problems for self-employment and household work activities. Accordingly, the WPAI was calculated by applying the overall work productivity loss for waged workers and activity impairment for all other participants. The costs of economic loss due to low productivity were estimated by multiplying the WPAI value obtained by surveying the patient with the standard wage for the corresponding gender and age [20].

As the total study period was 12 weeks, a discount rate was not applied. Moreover, an inflation rate of 0.46% for change in the consumer price index for medical care was applied to the costs estimated using the 2018 HIRA-NPS data to derive the costs for 2019. An exchange rate of 1156.4 won/dollars (as of 2019) was applied to derive values in US dollars [24].

### Economic perspective

Two perspectives were considered, and the primary analysis was conducted from a societal perspective, reflecting productivity loss due to neck pain. The societal perspective includes direct medical costs, direct non-medical costs, and costs associated with productivity loss. The analysis from a healthcare perspective included only direct medical costs and direct non-medical costs. Direct medical costs include the cost of intervention applied during the trial and the costs of informal care incurred during the trial period, such as the cost of treatment for neck pain from other Western medicine or KM institutions, the cost of analgesics, including over-the-counter drugs, and medical devices or exercise therapy. Direct non-medical costs include time and transportation costs.

### Data analysis

A primary analysis examining incremental cost utility from a societal perspective was performed to calculate the incremental cost-effectiveness ratio (ICER) by comparing the differential mean costs and QALY between the pharmacopuncture and usual care groups. ICERs were calculated by dividing the difference in total costs by the difference in effects.

Intention-to-treat (ITT) analysis was performed, and the missing values were imputed with multiple imputations (MI) using the Markov Chain Monte Carlo method and predictive mean matching. Twenty imputed datasets were generated; the covariates for imputation were treatment allocation, gender, age, and other correlated variables. Correlated variables (≥ 0.4) were included in the imputation model. The missing values for EQ-5D and SF-6D were 2–3%. The mice package in R version 4.0.1 was used for the imputation of missing values.

The significance level was set to 0.05 for all statistical analyses, and SAS 9.4 (© SAS Institute, Inc., Cary, NC, USA) and R studio 1.1.463 (© 2009–2018 RStudio, Inc., Boston, Massachusetts, United States) were used for the statistical analyses.

### Uncertainty

The uncertainty of ICER and costs was estimated using the bootstrap residual technique [17]. To obtain valid bootstrap inferences while dealing with multiple imputation, a total of 10,000 datasets were generated by applying 500 replications to 20 MI sets in accordance with Schomaker et al. [25], who recommended a bootstrap for each MI set. Residual bootstrapping was applied to the adjusted variables. The uncertainty of ICER was expressed as incremental cost-effect pairs (CE pairs) bootstrapped in cost-effectiveness planes (CE planes), and the percentage of pairs distributed in each quadrant was derived. Moreover, cost-effectiveness acceptability curves (CEACs), which indicate the probability of pharmacopuncture being cost-effective according to WTP, were derived. The threshold for willingness to pay was set at $26,374, based on the WTP amount survey result in Korea.

Three sensitivity analyses were conducted. First, a per-protocol analysis was performed only on patients who had received six or more treatments. Second, the method for calculating the productivity loss was altered. In the primary analysis, overall work impairment was calculated by including all patients and not just waged workers. Because it was assumed that only employed patients would have time and productivity loss in the sensitivity analysis, the loss of patients other than waged workers was processed as zero. Third, pharmacopuncture fees may vary from one hospital to another as they are not reimbursed. Accordingly, pharmacopuncture fees were assumed to be 1.5 and 2 times the pharmacopuncture cost in the primary analysis while performing the cost-effectiveness analysis. Finally, a cost-effectiveness analysis was performed on the Numerical rating scale(NRS) score for neck pain and Neck Disability Index (NDI). The VAS is a numeric pain scale that can be used for an objective assessment of subjective pain felt by patients. The patient selects the appropriate point along a 100 mm-long line with one end labeled 0, indicating no pain, and the other end labeled 10, indicating the worst pain possible [26]. The NDI is an assessment tool for disabilities caused by neck pain while performing daily activities. Ten items were rated on a 5-point scale for a total of 50 points, and the scores were converted to percentages, with higher scores indicating greater disability in daily living [27, 28]. The differences in NRS and NDI scores between the groups were assessed at baseline and 12 weeks after randomization, from which the differential NRS and differential NDI scores were calculated.

## Results

### Participants

Between September 2019 and June 2020, 263 patients were screened, of which 101 were randomly allocated to the pharmacopuncture and usual care groups. One participant from each group withdrew their consent before receiving the treatment; moreover, one participant from the PT group was dismissed by the investigator due to an administrative error in the random allocation process. Ultimately, 98 patients (49 each in the pharmacopuncture and usual care groups each) who received at least one treatment session were included in the ITT analysis. The flow chart of the study is shown in Supplementary Figure A2 (see Additional file 1). The patients’ baseline characteristics are shown in Supplementary Table A3 (see Additional file 1). At baseline, although there were no differences in pain and function, a significant difference was found in EQ-5D. The EQ-5D-5 L score of the pharmacopuncture group was 0.69 ± 0.13, which was significantly lower than that of the control group. There were no significant differences in SF-12 when analyzed by MCS and PCS or in SF6D. Meanwhile, the pharmacopuncture and usual care list used in this study are shown in Supplementary Table A4 (see Additional file 1).

### QALY

**Fig. 1:**
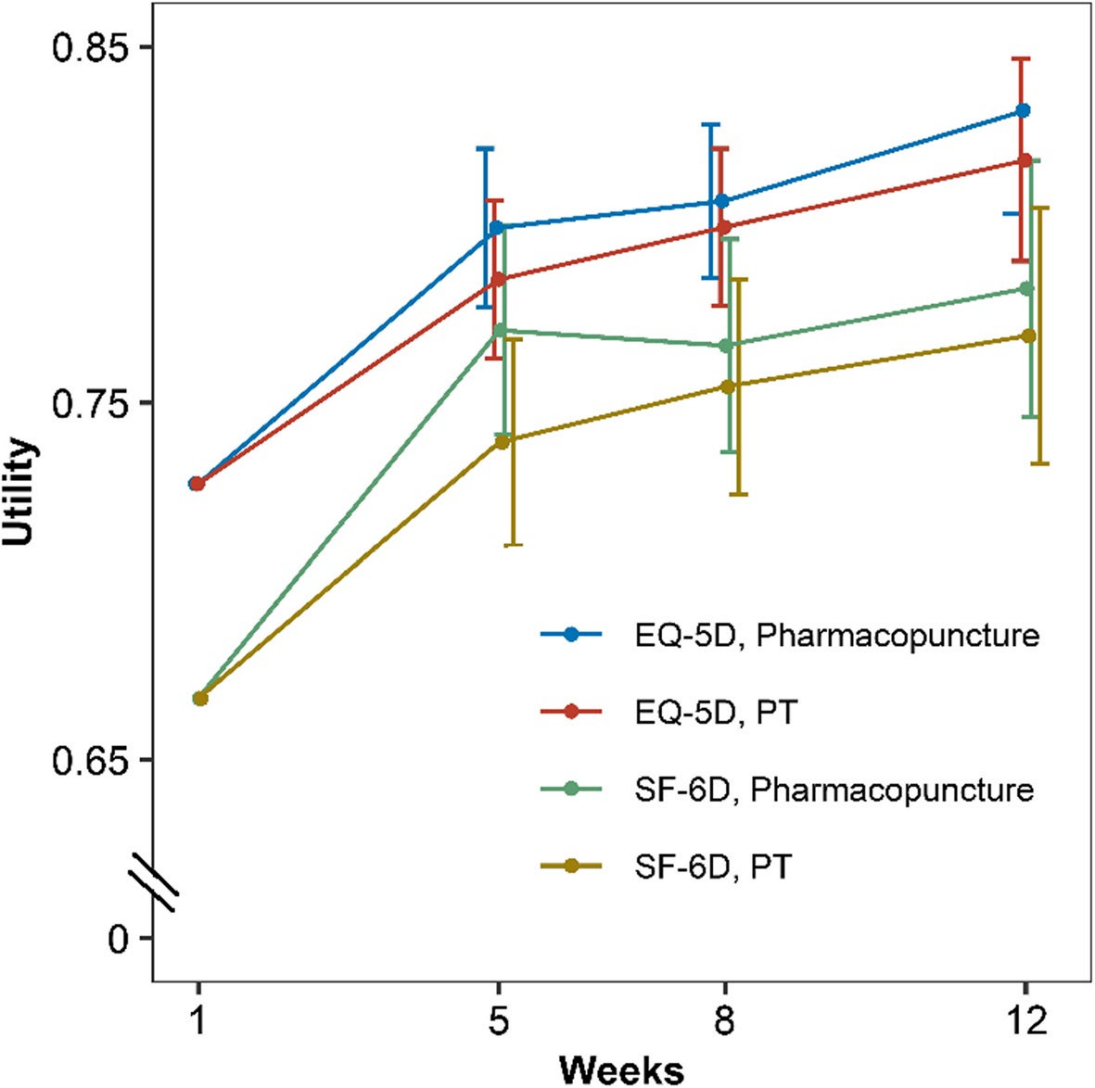
Distribution of utilities according to EQ-5D-5L and SF-6D by pharmacopuncture and usual care. *EQ-5D-5L : Questionnaire valuing health-related Quality of Life, SF-6D : An equipment evaluating health

The pharmacopuncture group showed a significantly lower EQ-5D-5 L score at baseline; however, the score was similar to those of the control group after 12 weeks. There were no significant differences between the two groups’ SF6D scores. The QALY was calculated using regression analyses adjusted for baseline values (Table 1; Fig. 1).

The adjusted QALY of the pharmacopuncture group for 12 weeks was 0.168 (95% confidence interval [CI], 0.165–0.171), while that of the control group was 0.166 (95% CI, 0.163–0.169). Accordingly, the adjusted differential QALY calculated based on the EQ-5D was 0.002. The adjusted QALY calculated based on SF-6D was 0.159 (95% CI, 0.155–0.164) in the pharmacopuncture group and 0.156 (95% CI, 0.151–0.160) in the control group, with an adjusted differential QALY of 0.003.

**Table 1:** Utility and quality of life years (QALY) after randomization by pharmacopuncture and usual care^a^

	Pharmacopuncture	Usual care	Difference	*P* value
	(*n* = 49)	(*n* = 49)		
**EQ5D**				
5th week	0.80 (0.78 to 0.82)	0.78 (0.76 to 0.81)	0.01 (-0.02 to 0.05)	0.374
8th week	0.81 (0.79 to 0.83)	0.80 (0.78 to 0.82)	0.01 (-0.02 to 0.04)	0.641
12th week	0.83 (0.80 to 0.86)	0.82 (0.79 to 0.85)	0.01 (-0.03 to 0.06)	0.501
QALY	0.168 (0.165 to 0.171)	0.166 (0.163 to 0.169)	0.002 (-0.003 to 0.007)	0.397
**SF6D**				
5th week	0.76 (0.73 to 0.79)	0.77 (0.74 to 0.80)	-0.01 (-0.05 to 0.04)	0.814
8th week	0.76 (0.73 to 0.79)	0.74 (0.71 to 0.77)	0.02 (-0.03 to 0.06)	0.419
12th week	0.76 (0.73 to 0.79)	0.75 (0.73 to 0.78)	0.01 (-0.03 to 0.05)	0.671
QALY	0.158 (0.154 to 0.163)	0.157 (0.153 to 0.161)	0.001 (-0.005 to 0.008)	0.717

Note. *P* < 0.05

^a^The total trial period was 12 weeks, and the primary endpoint was the 5th week. QALY was calculated using the trapezoidal rule. For the calculation of QALY, EQ-5D-5L was used in the primary analysis, and SF-6D (subsequently added) was used in the additional analysis. The values for each group and the differences are presented as baseline-adjusted least square estimates and 95% confidence intervals

### Costs

From a healthcare perspective, the combination of medical costs and direct non-medical costs were similar between the pharmacopuncture and control groups during the treatment period, whereas the cost was lower by $26 (95% CI, -123 to 64) in the pharmacopuncture group during the entire period. Regarding direct non-medical costs, the time cost was significantly lower in the pharmacopuncture group ($201; 95% CI, 186–217) than in the control group ($236; 95% CI, 210–263). Specifically, the time cost was significantly lower in the pharmacopuncture group because they spent less time being treated than the control group (Supplementary Table A5 (see Additional file 1). Moreover, the cost of productivity loss was also lower in the pharmacopuncture group than in the control group by $1,130 (95% CI, -1738 to -536); thus, the cost from a societal perspective was lower in the pharmacopuncture group by $1,157 (95% CI, -1868 to -573; Table 2). Specific details are provided in Supplementary Table A5 (see Additional file 1).

**Table 2:** Cost comparisons between pharmacopuncture and usual care^a^

	Pharmacopuncture	Usual care	Difference	*P*-value
	(*n* = 49)	(*n* = 49)		
**Medical costs**				
Intervention	234 (213 to 265)	200 (167 to 239)	34 (-13 to 78)	0.13
Total	254 (220 to 299)	247 (190 to 326)	7 (-74 to 79)	0.887
**Non-medical cost**				
Transportation	12 (8 to 18)	11 (6 to 18)	1 (-7 to 9)	0.719
Time loss for intervention	201 (186 to 217)	236 (210 to 263)	-35 (-66 to -5)	0.022
Total	214 (197 to 230)	247 (223 to 270)	-33 (-63 to -4)	0.024
**Healthcare perspectives**				
Intervention	448 (417 to 485)	446 (395 to 508)	1 (-65 to 62)	0.949
Total	468 (422 to 525)	494 (419 to 583)	-26 (-123 to 64)	0.593
**Productivity loss**				
Intervention	1,005 (874 to 1,140)	1,343 (1,194 to 1,504)	-338 (-543 to -134)	0.004
Total	2,253 (1,904 to 2,623)	3,384 (2,914 to 3,855)	-1,130 (-1,738 to -536)	0.002
**Societal perspectives**				
Intervention	1,453 (1,321 to 1,610)	1,790 (1,603 to 1,974)	-337 (-558 to -83)	0.008
Total	2,721 (2,340 to 3,122)	3,878 (3,437 to 4,411)	-1,157 (-1,868 to -573)	0.002

^a^The total trial period was 12 weeks. Intervention was up to the 5th week, during which time pharmacopuncture or physical therapy was applied.All variables were presented as means and at a 95% confidence interval. Confidence interval was estimated with bootstrap resampling The Korean won (KRW) was converted to the United States dollar (USD) at a conversion rate of 1 USD = 1,156 KRW.

**P < 0.05

***P < 0.001

### Cost-utility analysis

**Fig. 2:**
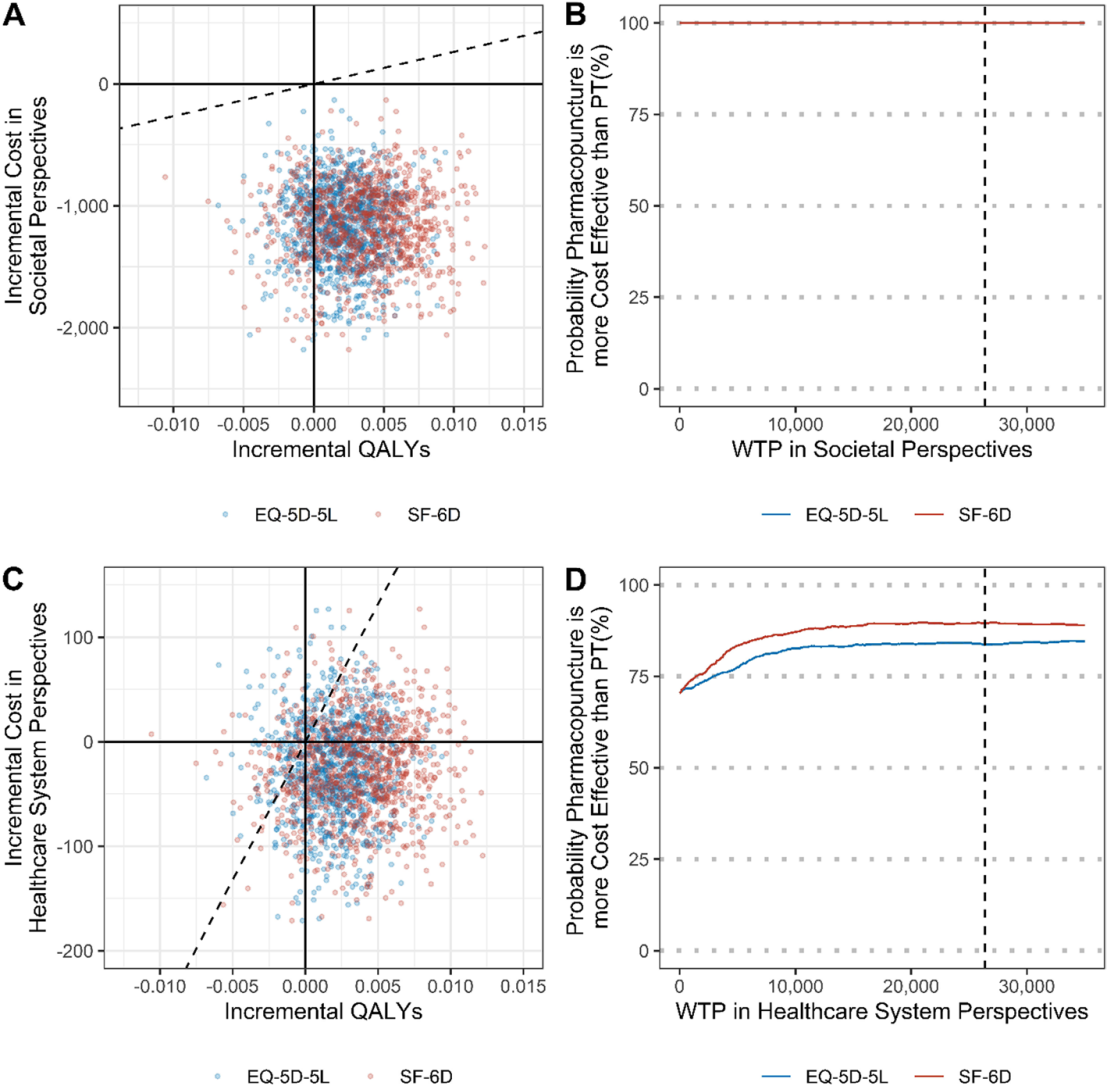
Cost-effectiveness plane and cost-effectiveness acceptability curves of pharmacopuncture compared with usual care

From a societal perspective, pharmacopuncture was superior as it showed a slightly higher QALY and a lower cost of $1,157 compared to usual care. Based on $26,375 as the WTP among the Korean public, the probability of being cost-effective from a societal perspective was 100% based on both EQ-5D and SF-6D (Table 3; Fig. 2).

An analysis from a healthcare perspective also showed a cost difference of $26, confirming that pharmacopuncture was better than usual care. Based on $26,375 as the WTP, the probability of cost-effectiveness from a societal perspective was 83.7% and 89.6% based on EQ-5D and SF-6D, respectively.

**Table 3:** The results of cost-effective analysis for pharmacopuncture compared with usual care

	Perspectives	QALY index	Difference in QALY	Difference in cost	ICER^a^ ($)	Probability^b^(%)
Main analysis	Societal	EQ-5D	0.002 (-0.003 to 0.007)	-1,157 (-1,868 to -573)	Dominant	100
		SF-6D	0.003 (-0.003 to 0.010)		Dominant	100
	Healthcare System	EQ-5D	0.002 (-0.003 to 0.007)	-26 (-123 to 64)	Dominant	83.7
		SF-6D	0.003 (-0.003 to 0.010)		Dominant	89.6
Sensitivity analysis 1	Societal	EQ-5D	0.002 (-0.003 to 0.007)	-1,149 (-1,812 to -540)	Dominant	100
	Healthcare System			-36 (-134 to 53)	Dominant	86.5
Sensitivity analysis 2	Societal	EQ-5D	0.002 (-0.003 to 0.007)	-1,011 (-1,822 to -274)	Dominant	100
	Healthcare System			-31 (-127 to 67)	Dominant	85
Sensitivity analysis 3	Societal	EQ-5D	0.002 (-0.003 to 0.007)	-1,090 (-1,726 to -488)	Dominant	100
	Healthcare System			40 (-51 to 127)	20,062	59.4
Sensitivity analysis 4	Societal	EQ-5D	0.002 (-0.003 to 0.007)	-1,023 (-1,670 to -359)	Dominant	99.9
	Healthcare System			107 (6 to 203)	53,197	25.4
Sensitivity analysis 5	Societal	NRS	1.27 (0.48 to 2.05)	-1,157 (-1,868 to -573)	Dominant	100
		NDI	4.75 (-0.14 to 9.65)		Dominant	100
	Healthcare System	NRS	1.27 (0.48 to 2.05)	-26 (-123 to 64)	Dominant	100
		NDI	4.75 (-0.14 to 9.65)		Dominant	97.7

For the base case analysis, QALY was calculated with EQ-5D in societal perspectives. The incremental cost was divided by the incremental QALY to calculate the incremental cost-effectiveness ratio (ICER). The distribution of cost and QALY was calculated by non-parametric bootstrap

In the main analysis, patients assigned to each group were followed-up up to 12 weeks, and missing values were imputed with multiple imputation. The costs from the healthcare system perspective include the costs of formal and informal healthcare involved in chronic neck pain treatment and the transportation and time costs. For the costs in the societal perspective, productivity costs from chronic neck pain are included

Sensitivity analysis. 1 was a per-protocol analysis, which included patients who received at least six treatment sessions (47 in the pharmacopuncture group and 45 in the physical therapy group). Sensitivity analysis 2. Assuming that only employed patients suffered income loss due to time and productivity losses. Sensitivity analysis 3. We applied $26 by multiplying 1.5 with the pharmacopuncture cost in the base case analysis. Sensitivity analysis 4. We applied $35 by multiplying 2 with the pharmacopuncture cost in the base-case analysis. Sensitivity analysis 5. Cost effectiveness analysis based on NRS and NDI

^a^If ICER has a negative value because pharmacopuncture is more cost-saving and effective, ICER is indicated as ‘dominant’

^b^Probability means probability of cost-effectiveness. They were calculated using the 1xWTP threshold ($26,375) and 1xGDP per capita ($31,838)

### Sensitivity analysis

The results of the sensitivity analysis are presented in Table 4. Concerning the cost-effectiveness plane in the analyses from each perspective, the probability of being distributed in the south quadrant, which is cost-saving and more effective, was 80.5% based on EQ-5D from a societal perspective.

**Table 4:** Results of uncertainty analysis

	Perspectives	QALY index	Probability of cost-effectiveness by cost-effectiveness plane (%)				Incremental net benefit ($)
			Cost-saving + More effective	Cost-increasing + More effective	Cost-saving + Less effective	Cost-increasing + Less effective	
Main analysis	Societal	EQ-5D	80.5	0	19.5	0	1,209 (627 to 1,925)
		SF-6D	85.3	0	14.7	0	1,249 (654 to 1,965)
	Healthcare System	EQ-5D	57.1	23.4	13.3	6.2	80 (-85 to 234)
		SF-6D	58.8	26.5	11.6	3.1	120 (-58 to 310)
Sensitivity analysis 1	Societal	EQ-5D	81.3	0	18.7	0	1,213 (586 to 1,873)
	Healthcare System		62.7	18.6	14.3	4.4	90 (-61 to 239)
Sensitivity analysis 2	Societal	EQ-5D	80.2	0.3	19.5	0	1,072 (322 to 1,909)
	Healthcare System		58.7	21.8	13.5	6	84 (-73 to 244)
Sensitivity analysis 3	Societal	EQ-5D	80.5	0	19.5	0	1,150 (542 to 1,782)
	Healthcare System		16.3	64.2	3.4	16.1	15 (-151 to 173)
Sensitivity analysis 4	Societal	EQ-5D	80.4	0.1	19.5	0	1,078 (402 to 1,760)
	Healthcare System		1.6	78.9	0.2	19.3	-54 (-218 to 109)
Sensitivity analysis 5	Societal	NRS	100	0	0	0	4,544 (2,444 to 6,825)
		NDI	97.5	0	2.5	0	2,368 (1,049 to 3,827)
	Healthcare System	NRS	70.4	29.6	0	0	3,415 (1,478 to 5,414)
		NDI	68.8	28.7	1.6	0.9	1,238 (21 to 2,494)

For the base case analysis, QALY was calculated with EQ-5D in societal perspectives. The distribution of cost and QALY was calculated by non-parametric bootstrap. The incremental net benefit (INB) was calculated using the 1xWTP threshold ($26,375) and 1xGDP per capita ($31,838)

In the main analysis, patients assigned to each group were followed-up up to 12 weeks, and missing values were imputed with multiple imputation. The costs from the healthcare system perspective include the costs of formal and informal healthcare involved in chronic neck pain treatment and the transportation and time costs. For the costs in the societal perspective, productivity costs from chronic neck pain are included.

Sensitivity analysis. 1 was a per-protocol analysis, which included patients who received at least six treatment sessions (47 in the pharmacopuncture group and 45 in the physical therapy group). Sensitivity analysis 2. Assuming that only employed patients suffered income loss due to time and productivity losses. Sensitivity analysis 3. We applied $26 by multiplying 1.5 with the pharmacopuncture cost in the base case analysis. Sensitivity analysis 4. We applied $35 by multiplying 2 with the pharmacopuncture cost in the base-case analysis. Sensitivity analysis 5. Cost effectiveness analysis based on NRS and NDI.

First, a cost-effectiveness analysis was performed on patients who had received at least six treatment sessions. The difference in costs was $1,149 from a societal perspective and $36 from a healthcare perspective. On the CE plane, CE pairs showed the highest distribution of 81.3% in the SE quadrant; thus, the probability of pharmacopuncture being more cost-effective from a societal perspective was 100% based on QALY.

## Discussion

This economic evaluation was performed based on an RCT that compared pharmacopuncture and usual care for chronic neck pain. Compared to the usual care group, the pharmacopuncture group had higher intervention costs, lower direct non-medical costs, and lower indirect costs (costs due to productivity loss). Consequently, the costs from a societal perspective were significantly lower in the pharmacopuncture group during the entire study period (12 weeks), whereas there were no significant differences in the costs from a healthcare perspective. The pharmacopuncture group tended to show a slightly higher QALY based on EQ-5D and SF-6D, although the differences between two groups were not significant. However, the costs were lower in the pharmacopuncture group. Therefore, ICER analysis was dominant, indicating that pharmacopuncture is more cost-effective from both the societal and healthcare perspectives. Sensitivity analyses with the pharmacopuncture fee set at 1.5 and 2.0 times showed higher costs in the pharmacopuncture group from a healthcare perspective. The ICER was lower than the WTP when the fee was set to 1.5, which confirmed that it is an alternative that is acceptable from a societal perspective not only for base cases but also when the fee is increased by a factor of 1.5. When the pharmacopuncture fee was increased by two times, the costs were still lower from a societal perspective, while the probability of pharmacopuncture being cost-effective was higher.

In the sensitivity analysis of this study, not only cost-utility analysis but also cost-effectiveness analysis was performed. Costs were compared with clinical outcomes (NRS score for neck pain and NDI). In many cases of economic evaluation, costs are compared to the QALY based on utility. However, utility is broad in nature and therefore does not accurately reflect disease-specific health status and is relatively insensitive to detecting small changes [29]. For chronic pain disorders such as the target disease in the present study that do not significantly affect basic daily life activities such as walking, dressing, and washing, it is difficult to reflect the changes based on utility measured by EQ-5D; thus, cost-effectiveness analysis results were presented together. As confirmed in published RCT results on effectiveness, there were no significant differences between the two groups regarding functions measured by NDI or NPQ, or regarding pain measured by NRS or VAS. Furthermore, there were no significant differences in EQ-5D and SF-6D scores. The differential QALY calculated using EQ-5D and SF-6D was very small (0.001–0.002) and not statistically significant. Accordingly, in keeping with the recommendation of using other outcomes that are more sensitive [29], the present study used QOL as well as pain and functional impairment indicators to perform additional cost-effectiveness analyses.

Meanwhile, there was little difference in QALY between the two groups when based on the EQ-5D and SF-6D. Both the EQ-5D and SF-6D measure quality of life, but there are minor differences; SF-6D focuses more on social functioning, whereas EQ-5D focuses on physical functioning ^46^. The fact that there is no difference between the two instruments shows that pharmacopuncture improved various aspects of the quality of life of patients with chronic neck pain.

Direct medical costs were higher in the pharmacopuncture group due to the difference in costs between pharmacopuncture and PT. Medical costs, other than the intervention costs, during the trial were higher in the usual care group, indicating that they had more hospital visits for treatment of neck pain in addition to the treatment in the protocol. Among direct non-medical costs, time cost was significantly lower in the pharmacopuncture group, which was due to the length of the pharmacopuncture treatment being shorter than the usual care time. Compared to PT, which requires more than a certain amount of time, pharmacopuncture is completed with needling; thus, the time required for the treatment is very short. The results showed that pharmacopuncture had a superior effect despite the relatively short procedure time.

The indirect costs estimated by the productivity loss score were significantly lower in the pharmacopuncture group, and the difference between the two groups was relatively large. In the primary analysis performed in the present study, productivity loss among waged workers was calculated as overall work productivity, considering both absenteeism and presenteeism. Many economic evaluation studies, including those on chronic neck pain, generally use absenteeism to calculate costs [30–32]. However, productivity loss related to pain occurs mostly at work [33]; thus, utilizing only absenteeism could lead to an underestimation of productivity loss [32]. In particular, chronic neck pain characteristically has a high likelihood of affecting work efficiency. Few patients were absent in the present study. Accordingly, this study also considered presenteeism to estimate a more suitable productivity loss due to chronic neck pain. The study also calculated productivity loss by considering activity impairment among not only waged workers but also non-waged workers such as homemakers and self-employed individuals. This was because the percentage of non-wage workers was not small, and calculating their productivity loss and opportunity costs would be more reasonable [34].

This economic evaluation offered the benefit of being conducted alongside a well-designed, pragmatic RCT. The pragmatic approach is a design for overcoming the limitations of traditional explanatory RCTs, which could increase the generalizability of the findings by carrying out the trial in an environment closer to the real world and contributing to actual clinical and policy decision making [35, 36]. Therefore, pragmatic design is the most suitable for economic evaluation [32]. When estimating the costs, a patient questionnaire survey, micro-costing, and health insurance claims data were used in consideration of both the accuracy and the data’s generalizability. In the present study, the interval between outcome measures was short, and costs were investigated during each visit to minimize recall bias that could occur while using patient response data [32].

This study investigated validity indicators and cost expenditures over 12 weeks of follow-up (F/U). This short time horizon is the present study’s limitation. The results of this study couldn’t show the long-term effect of pharmacopuncture on the quality of life of patients with chronic cervical pain. Longer follow-up period studies or modeling studies should be performed in the future. To the best of our knowledge, there have been no economic evaluations of pharmacopuncture for chronic neck pain. While there have been some studies on acupuncture being more cost-effective than usual care [30, 37], there were no previous studies that could be compared and tested against the present study. Therefore, additional studies are needed to strengthen the evidence for the findings of the present study.

## Conclusions

Pharmacopuncture is more cost-effective than the usual care for chronic neck pain. The same results were found in various sensitivity analyses. The current study’s findings provide useful information to clinicians and policy makers who are considering new treatment options for neck pain. Additional studies are needed to strengthen the evidence supporting the findings of the present study.

### Supplementary Information

**Additional file 1: Supplementary Table A1.**Schedule of the participants**. Supplementary Table A2. **The cost calculation method, the associated data source, and unit cost*.**Supplementary Table A3.** Demographic and clinical characteristics of the patients at baseline^*^. **Supplementary Table A4. **The list of treatment provided to patients during the treatment period. **Supplementary Table A5.** Healthcare costs and resource uses after randomization by pharmacopuncture and physical therapy^*^. **Supplementary Figure A1.** Flow chart of the study

## Data Availability

Patient samples can be obtained via the HIRA website by completing the End User Agreement for Patient Samples. Patient samples are provided in a DVD (text file) format and a fee for the samples may be charged. See https://opendata.hira.or.kr/home.do.

## Abbreviations

QALY: Quality-adjusted life year
WTP: Willingness-to-pay
YLD: Years lived with disability
CAM: Complementary and alternative medicine
KM: Korean medicine
PT: Physical therapy
VAS: Visual analog scale
QOL: Quality of life
EMR: Electronic medical records
CRF: Case report form
HIRA: Health Insurance Review and Assessment’s
AUC: Area under the curve
NPS: National Patient Samples
WPAI: SHP-Work Productivity and Activity Impairment-Specific Health Problem
ICER: Incremental cost-effectiveness ratio
ITT: Intention-to-treat
MI: Multiple imputations
CE pairs: Cost-effect pairs
CE planes: Cost-effectiveness planes
CEACs: Cost-effectiveness acceptability curves
NRS: Numerical rating scale
