# Supplementary material for: An economic evaluation of pharmacopuncture versus usual care for chronic neck pain: a pragmatic randomized controlled trial
Source: BMC Health Serv Res. 2023 Nov 23;23:1286. doi: 10.1186/s12913-023-10325-w (PMC10666315; doi:10.1186/s12913-023-10325-w)
Supplement: Supplementary file 1 — Additional file 1: Supplementary Table A1.Schedule of the participants. Supplementary Table A2. The cost calculation method, the associated data source, and unit cost*.Supplementary Table A3. Demographic and clinical characteristics of the patients at baseline*. Supplementary Table A4. The list of treatment provided to patients during the treatment period. Supplementary Table A5. Healthcare costs and resource uses after randomization by pharmacopuncture and physical therapy*. Supplementary Figure A1. Flow chart of the study [file 12913_2023_10325_MOESM1_ESM.docx]

| **Supplementary Table A1.** Schedule of the participants | | | | | | | | | |
| --- | --- | --- | --- | --- | --- | --- | --- | --- | --- |
| Time point (week) | Screening | Enrollment allocation | Active treatment after allocation | | | | Follow-up | | |
|  | -1 | 0 (Baseline) | 1 | 2 | 3 | 4 | 5 (PEO) | 8 | 12 |
| Visit window |  | **± 3** | **± 3** | **± 3** | **± 3** | **± 3** | **± 3** | **± 7** | **± 7** |
| **Enrollment** | | | | | | | | | |
| Eligibility screening | ○ | ○ | – | – | – | – | – | – | – |
| Written informed consent | ○ | – | – | – | – | – | – | – | – |
| Vital signs | ○ | ○ | ○ | ○ | ○ | ○ | ○ | ○ | ○ |
| Sociodemographic characteristics, medical history  (e.g., neck pain, medication history) | ○ | – | – | – | – | – | – | – | – |
| Cervical spine X-ray | ○ | – | – | – | – | – | – | – | – |
| Randomized allocation | – | ○ | – | – | – | – | – | – | – |
| **Interventions** | | | | | | | | | |
| Treatment for the pharmacopuncture group  (experimental group) |  |  | ← 2 (1-3) times/week → | | | |  |  |  |
| Treatment for the PT group  (control group) |  |  | ← 2 (1-3) times/week → | | | |  |  |  |
| **Assessments** | | | | | | | | | |
| Symptoms and change | – | – | ○ | ○ | ○ | ○ | ○ | ○ | ○ |
| NRS scores for neck and arm pain | – | – | ○ | ○ | ○ | ○ | ○ | ○ | ○ |
| VAS score for neck pain | ○ | ○ | ○ | ○ | ○ | ○ | ○ | ○ | ○ |
| VAS score for arm pain | – | – | ○ | ○ | ○ | ○ | ○ | ○ | ○ |
| NPQ | – | – | ○ | – | – | – | ○ | ○ | ○ |
| NDI | – | – | ○ | – | – | – | ○ | ○ | ○ |
| PGIC | – | – | – | – | – | – | ○ | ○ | ○ |
| SF-12 | – | – | ○ | – | – | – | ○ | ○ | ○ |
| EQ-5D-5L | – | – | ○ | – | – | – | ○ | ○ | ○ |
| Physical examination | – | – | ○ | – | – | – | ○ | – | – |
| Drug use | ○ | ○ | ○ | ○ | ○ | ○ | ○ | ○ | ○ |
| Evaluation of medical costs | – | – | ○ | – | – | – | ○ | ○ | ○ |
| Evaluation of time cost |  |  |  | ○ |  |  |  |  |  |
| Evaluation of lost work productivity |  |  | ○ | ○ | ○ | ○ | ○ | ○ | ○ |
| Credibility and expectancy |  |  | ○ |  |  |  |  |  |  |
| Blood analysis |  |  | ○ |  |  |  | ○ |  |  |
| Adverse events |  | ○ | ← Every visit → | | | | ○ | ○ | ○ |
| ***PT***, physical therapy; ***NRS***, numeric rating scale; ***VAS***, visual analogue scale; ***NPQ***, Northwick Park Questionnaire; ***NDI***, neck disability index; ***PEO,*** Primary end point; ***PGIC***, patient global impression of change, ***SF-12***, 12-Item Short Form Health Survey; ***EQ-5D-5L***, EuroQoL 5-dimension 5-level instrument. | | | | | | | | | |

**Supplementary Table A2.** The cost calculation method, the associated data source, and unit cost*

|  | | | |
| --- | --- | --- | --- |
| Type of costs | Calculation method | Source of the original data | Unit cost |
| Pharmacopuncture ^†^ | Pharmacopuncture is a non-covered treatment. The costs commonly prescribed by medical institutions were investigated and applied. | Survey | $17 |
| Physical therapy (Electrotherapy)^†^ | All details of the actual physical therapy performed on the patient are recorded, and the price of the technique corresponding to the HIRA price index is applied. | HIRA price index 2019 | $5  [1 – 9] |
| Physical therapy (Extracorporeal Shock Wave Therapy, ESWT)^†^ | ESWT is a non-covered treatment. The costs commonly prescribed by medical institutions were investigated and applied. | Survey | $69 |
| Consultation fee at first visit by Korean traditional medicine^‡^ | When a patient visits a medical institution, a medical doctor conducts an examination. The consultation fee is set differently for the first and recurrent visits, and it also differs between Korean medicine doctors and general practitioners. The pharmacopuncture therapy group was seen by a Korean medicine doctor and the usual care group by a general practitioner. | HIRA price index 2019 | $12 |
| Consultation fee at recursive visit by Korean traditional medicine^‡^ |  | HIRA price index 2019 | $8 |
| Consultation fee at first visit by Western medicine^‡^ |  | HIRA price index 2019 | $15 |
| Consultation fee at recursive visit by Western medicine^‡^ |  | HIRA price index 2019 | $11 |
| Syndrome differentiation technique fee^‡^ | Korean traditional medicine has a system that examines the comprehensive symptoms of a patient based on unique theories and experiences. This is called syndrome differentiation (辨證), and all Korean medicine doctors may charge an additional syndrome differentiation technique fee (辨證技術料) according to the examination. This can be charged once a week, and the patient can visit up to twice a week during the 5-week intervention period. | HIRA price index 2019 | $3 |
| X-ray^†^ | On the patient's first visit, an X-ray examination of the cervical area was performed twice. | HIRA price index 2019 | $8 |
| Over the counter^¶^ | If a patient purchases a drug over the counter, the actual amount spent is surveyed. | Patient survey | $3  [3 - 3] |
| Additional private Korean Traditional Medicine outpatient visits^**^ | The details of the patient's personal visits to medical institutions other than the clinical trial site and the associated expenses were surveyed. In this case, the amount indicated in the patient’s response was a copayment, and the reimbursement was unknown. Accordingly, the reimbursement was calculated from the claim data of chronic neck pain patients in the Health Insurance Review and Assessment Service – National Patient Sample (HIRA-NPS) in 2018. The calculated average reimbursement was stratified by the patient’s gender and age and matched. | Patient survey  2018 HIRA-NPS | $41  [23 – 57] |
| Additional private Western medicine outpatient visits^**^ |  |  | $60  [26 – 133] |
| Exercise, massage, etc.^††^ | The cost and number of services purchased by the patient were surveyed. | Patient survey | $25  [3 – 199] |
| Transportation^‡‡^ | The actual transportation cost to visit the clinical trial site was surveyed one week after the baseline, and this was multiplied by the actual number of visits. | Patient survey | $3  [1 – 17] |
| Time cost^§§^ | After one week from baseline, all of the time taken for the patient from leaving the house, going to the hospital, interviewing, waiting, treatment, and returning home was surveyed. This was multiplied by the actual number of visits. | Patient survey | — |
| Productivity cost^§§^ | The productivity loss was assessed using Work Productivity and Activity Impairment – Specific Health Problem (WPAI-SHP). Then, according to the human capital approach, the productivity loss was multiplied by gender and age-stratified income to calculate the income loss due to the productivity loss, which is regarded as the productivity cost. In the base case analysis, overall work impairment was applied to the productivity loss of employed patients, and activity impairment was applied to unemployed patients. The result of estimating the productivity costs only for employed patients alone is presented in the section on sensitivity analysis. | Patient survey | — |
| Income^§§^ | Gender- and age-stratified income was applied. | 2019 survey report on labor conditions by employment type | — |
| * If there is no difference in the calculated costs for each patient, only the unit cost is presented, and if there is a patient-specific difference in the costs, the mean [min - max] of the calculated costs is indicated. In the case of applying a uniform cost, only the unit cost is presented. If costs are set in a patient-specific manner, the mean [min - max] of the calculated costs is indicated.  † Cost for each instance of the examination/treatment session  ‡ Cost for each visit to the clinical trial institution  § Cost for each instance of prescription  ¶ Cost for each OTC purchase  ** Cost for each visit to the medical institution  †† Cost for the one-time use of the service  ‡‡ Cost for each visit to the clinical trial institution  §§ For these costs, the costs expended by the patients were not directly surveyed, and associated costs were calculated indirectly by considering the patients’ income. Therefore, the unit costs are not presented in these cases. | | | |

| **Supplementary Table A3.** Demographic and clinical characteristics of the patients at baseline^*^ | | | |
| --- | --- | --- | --- |
|  | Pharmacopuncture | Usual care | p-value |
|  | (n = 49) | (n = 49) |  |
| **Sex** |  |  |  |
| Female | 34 (69.4) | 35 (71.4) | 1 |
| Male | 15 (30.6) | 14 (28.6) |  |
| **Age** | 49.6 ± 12.2 | 47.7 ± 9.9 | 0.39 |
| **Height** | 163.2 ± 7.9 | 163.5 ± 8.3 | 0.817 |
| **Body weight** | 65.1 ± 12.4 | 64.5 ± 11.2 | 0.798 |
| **BMI** | 24.4 ± 4.2 | 24.0 ± 3.3 | 0.633 |
| **Duration of neck pain (months)** | 28.5 ± 33.3 | 28.4 ± 23.4 | 0.983 |
| **Previous medical use^†^** |  |  |  |
| Yes | 21 (42.9) | 22 (44.9) | 1 |
| No | 28 (57.1) | 27 (55.1) |  |
| **VAS^‡^** |  |  |  |
| Neck | 63.9 ± 11.0 | 65.3 ± 10.7 | 0.529 |
| Arm | 42.0 ± 27.8 | 37.2 ± 29.7 | 0.411 |
| **NRS^§^** |  |  |  |
| Neck | 6.4 ± 1.1 | 6.6 ± 1.1 | 0.471 |
| Arm | 4.1 ± 2.8 | 3.9 ± 3.0 | 0.727 |
| **NPQ^‖^** | 43.5 ± 13.1 | 41.0 ± 10.1 | 0.291 |
| **NDI^¶^** | 36.4 ± 12.7 | 32.8 ± 9.1 | 0.105 |
| **EQ-5D-5L score^**^** | 0.69 ± 0.13 | 0.76 ± 0.10 | 0.004 |
| **SF-12 score^††^** |  |  |  |
| MCS | 38.7 ± 8.4 | 40.5 ± 7.9 | 0.275 |
| PCS | 45.5 ± 9.3 | 48.0 ± 9.7 | 0.185 |
| **SF6D^‡‡^** | 0.65 ± 0.11 | 0.69 ± 0.11 | 0.089 |
| **WPAI^§§^** | 56.5 ± 18.1 | 59.9 ± 12.9 | 0.287 |
| * Data are represented as either mean ± standard deviation (SD) or number (%). Values of continuous variables between the two groups were compared using independent t-tests, and values of categorical variables were compared using the chi-square test or Fisher’s exact test. | | | |
| † Any medical intervention sought by the patient within the last 3 months for the purpose of alleviating neck pain | | | |
| ‡ The visual-analogue scale score for pain was measured by having patients indicate their own pain level on a line from 0 (*no pain*) to 100 (*most severe pain imaginable*) in millimeters. | | | |
| § The numeric rating scale score for pain was measured by having patients report their own pain level as a number from 0 (*no pain*) to 10 (*most severe pain imaginable*). | | | |
| ‖ The Neck Disability Index score was calculated as a percentage, where higher scores indicate more severe disability. | | | |
| ¶ The Northwick Park Questionnaire score was calculated as a percentage, where higher scores indicate more severe pain and disability. | | | |
| ** The five-level EuroQol 5 Dimension score was calculated by converting patient responses to a scale from -0.066 (*lowest quality of life*) to 1 (*highest quality of life*). | | | |
| †† The Medical Outcomes Study 12-Item Short-Form General Survey score was calculated by converting patient responses to a scale from 0 (*lowest quality of life*) to 100 (*highest quality of life*). | | | |
| ‡‡ The six-dimensional health state short form was calculated using the method of Brazier et al. (2004). The range was from 0 (*lowest quality of life*) to 1 (*highest quality of life*). | | | |
| §§ The Work Productivity and Activity Impairment Questionnaire: Specific Health Problem score was calculated as a percentage, evaluating the overall work impairment due to neck pain during the last week. For patients who were unemployed or not working in the last week, the activity impairment was rated. | | | |

| **Supplementary Table A4.** The list of treatment provided to patients during the treatment period | | |
| --- | --- | --- |
| Interevention | N (%) | Mean ± SD |
| **Pharmacopuncture group** **(n = 49)** |  |  |
| Shinbaro 2 | 47 (95.9) | 8.4 ± 3.0 |
| Harpagophytum procumbens | 17 (34.7) | 7.2 ± 1.8 |
| Shinbaro 1 | 2 (4.1) | 8 ± 0 |
| **PT group** **(n = 49)** |  |  |
| Interferential Current Therapy | 36 (73.5) | 7.4 ± 1.7 |
| Deep heat therapy | 31 (63.3) | 6.0 ± 3.2 |
| Superficial heat therapy | 13 (26.5) | 7.9 ± 0.3 |
| Laser therapy | 13 (26.5) | 6.4 ± 1 |
| Transcutaneous electrical nerve stimulation | 12 (24.5) | 6.5 ± 2.2 |
| Extracorporeal Shock Wave Therapy | 6 (12.2) | 4.3 ± 2.4 |
| The number (%) of each treatment provided to patients of both groups during the treatment period are shown in this table. Multiple use were allowed. **PT**, physical therapy | | |

| **Supplementary Table A5.** Healthcare costs and resource uses after randomization by pharmacopuncture and physical therapy^*^ | | | | |
| --- | --- | --- | --- | --- |
|  | Pharmacopuncture (n=49) | | Physical therapy (n=49) | |
|  | Volume | cost ± SD | Volume | cost ± SD |
| **Pharmacopuncture** (49/49) | 49 (100.0) | 160 ± 24 | — | — |
| **Physical therapy** (49/49) | — | — | 49 (100.0) | 87 ± 128 |
| **Examination** (49/49) | 49 (100.0) | 84 ± 10 | 49 (100.0) | 97 ± 17 |
| **Travel cost** (47/46) | — | 13 ± 20 | — | 11 ± 21 |
| **Time cost** (47/46)^†^ |  |  |  |  |
| Time cost for travel | 501.7 ± 269.6 | 131 ± 67 | 606.4 ± 443.5 | 176 ± 124 |
| Time cost for waiting | 77.7 ± 49.7 | 22 ± 15 | 59.8 ± 26.7 | 18 ± 11 |
| Time cost for treatment | 112.2 ± 74.5 | 30 ± 20 | 201.8 ± 89.3 | 60 ± 30 |
| **Total number of interventions received** (49/49) | 7.7 ± 1.1 | — | 7.6 ± 1.5 | — |
| **Additional private healthcare usage** |  |  |  |  |
| **OTC** (49/49) | 0 (0.0) | 0 ± 0 | 1 (2.1) | 0 ± 0 |
| **Additional private Korean Traditional Medicine**  **outpatient visits** | |  |  |  |
| Intervention (48/48) | 1 (2.1) | 1 ± 7 | 1 (2.1) | 1 ± 4 |
| Total (49/49) | 3 (6.2) | 18 ± 113 | 4 (8.3) | 33 ± 164 |
| **Additional private Western medicine outpatient visits** | |  |  |  |
| Intervention (48/48) | 2 (4.2) | 15 ± 96 | 3 (6.2) | 6 ± 34 |
| Total (49/49) | 2 (4.2) | 15 ± 96 | 5 (10.4) | 11 ± 40 |
| **Etc.** (49/49) | 7 (14.6) | 4 ± 16 | 13 (27.1) | 18 ± 46 |
|  | | | | |
| * Prices are expressed as mean ± SD KRW (Korean Won) and were converted to USD (United States Dollar); 1 USD was calculated at 1,156 KRW.  † The unit is in “minutes.” | | | | |


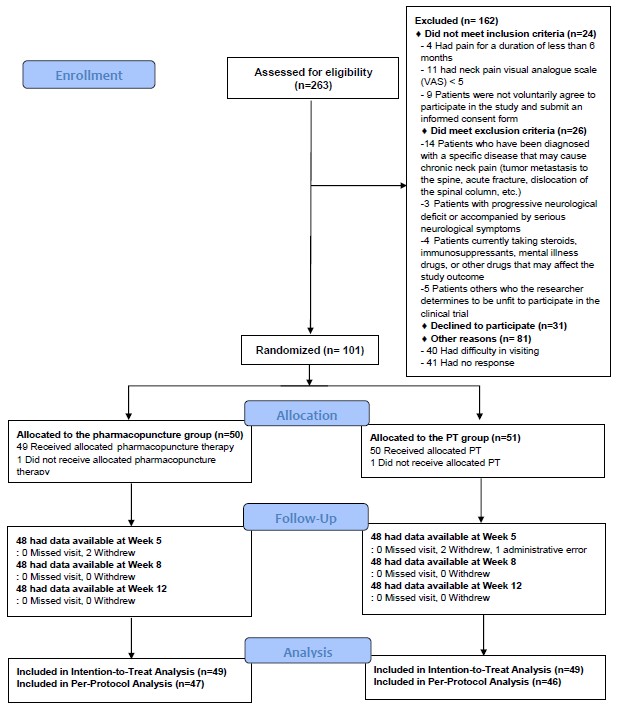


**Supplementary Figure A1.** Flow chart of the study
